# High expression of DJ‐1 promotes growth and invasion via the PTEN‐AKT pathway and predicts a poor prognosis in colorectal cancer

**DOI:** 10.1002/cam4.1325

**Published:** 2018-02-14

**Authors:** Yong Lin, Qian Chen, Quan‐xing Liu, Dong Zhou, Xiao Lu, Xu‐feng Deng, Hua Yang, Hong Zheng, Yuan Qiu

**Affiliations:** ^1^ Department of Pathology The first affiliated Hospital Third Military Medical University Chongqing 400037 China; ^2^ Department of Thoracic Surgery Xinqiao Hospital Third Military Medical University Chongqing 400037 China; ^3^ Department of Cardiothoracic Surgery First People's Hospital of Zunyi Guizhou 563000 China; ^4^ Department of General Surgery Xinqiao Hospital The Third Military Medical University Chongqing 400037 China

**Keywords:** Colorectal cancer, DJ‐1, invasion, proliferation, PTEN

## Abstract

Cancer cell invasion and unlimited proliferation are key factors in patients with colorectal cancer (CRC). Increased protein deglycase DJ‐1 in cancer cells is known to promote tumor growth; however, its role in CRC progression is not well defined. In this study, we investigated 100 CRC patients with disease stages I–IV to determine whether DJ‐1 could serve as a prognostic biomarker in CRC. These results showed that DJ‐1 expression in CRC tissues was higher than that in normal colon tissues and was associated with the (Tumor Node Metastasis) TNM stage. CRC patients with low DJ‐1 expression had a longer overall survival than those with high expression, and multivariate and univariate analyses indicated that DJ‐1 expression was an independent prognostic factor for overall survival in CRC. Furthermore, DJ‐1 overexpression in two colon cancer cell lines, HCT116 and SW480, activated protein kinase AKT and downregulated tumor suppressor PTEN, whereas DJ‐1 knockdown upregulated PTEN expression and effectively suppressed CRC cell invasion and proliferation both in vitro and in vivo, revealing a mechanism underlying DJ‐1 pro‐oncogenic activity in CRC. Treatment of MK2206, the specific AKT inhibitor, significantly decreased DJ‐1‐mediated cell proliferation and mobility in vitro. Taken together, these results suggest that DJ‐1 may be a novel prognostic biomarker and potential therapeutic target in human CRC.

## Introduction

Colorectal cancer (CRC) is the second and third most common type of human malignancy in women and men, respectively, causing 700,000 deaths annually worldwide 1. Although the CRC mortality rate has been dramatically decreased owing to significant improvement in early diagnostics, surgical techniques, and chemotherapy, the disease‐free survival rate for patients with advanced‐stage CRC remains low. Because invasion and proliferation are the two most important characteristics of CRC contributing to a poor prognosis 2, it is imperative to identify novel therapeutic targets and develop more effective treatment strategies for patients with CRC.

Protein deglycase DJ‐1, which is the cause of autosomal recessive early‐onset familial Parkinson's disease 3, was first identified as an oncogene in cooperation with H‐RAS and caused malignant transformation of mouse NIH3T3 cells 4. Moreover, DJ‐1 has been found in various cancerous tissues, including medulloblastoma 5, glioma 6, lung cancer 7, and oral squamous carcinoma 8. Abundant DJ‐1 was shown to promote the growth and multidrug resistance of tumor cells 9 by downregulating the expression of bax and inhibiting caspase activation 10. On the other hand, DJ‐1 was reported to inhibit melanoma metastasis into the lung by decreasing the levels of growth hormone 11, suggesting that the role of DJ‐1 in tumor progression is controversial. However, there is no information concerning the functional and clinical value of DJ‐1 in CRC. Therefore, it is necessary to explore the precise role of DJ‐1 in human CRC.

As described here, we analyzed the expression and prognostic significance of DJ‐1 using CRC clinical samples and explored the relationship between DJ‐1 expression and cancer cell malignancy in two colon cancer cell lines. We found that DJ‐1 is differentially expressed in CRC low‐ and high‐risk samples; the different CRC tissue expression of DJ‐1 is also reflected at the TNM stage, suggesting its possible role as a clinical prognostic target. Furthermore, DJ‐1 promoted the invasion and proliferation of CRC cells both in vitro and in vivo after implantation into nude mice by inhibiting tumor suppressor PTEN and activating protein kinase AKT. Taken together, our results suggest that DJ‐1 may be a novel prognostic marker and a potential therapeutic target in human CRC.

## Materials and Methods

### Clinical samples

Tumor tissues and corresponding tumor adjacent tissue specimens were obtained from 100 patients with CRC diagnosed and operated at Xinqiao Hospital, the Third Military Medical University, from 2006 to 2008, and followed up to 2014. None of the patients had received radiotherapy or chemotherapy before surgery. All patients provided informed consent to use their tissues for research purposes.

### Cell culture and treatment

Colon cancer cell lines SW480 and HCT116 were obtained from the ATCC and were maintained in a humidified atmosphere of 5% CO_2_ at 37°C in DMEM and supplemented with 10% fetal bovine serum (FBS) (HyClone Logan, Utah) and penicillin:streptomycin (1:1000) (Life Technologies Waltham, MA). When needed, cells were treated with a specific AKT inhibitor, MK2206 (CST Danvers, MA), added at 1 *μ*mol/L for 48 h.

### Overexpression and stable knockdown of DJ‐1 in cancer cells

To obtain DJ‐1 overexpressing cells, the CRC cells were transfected with the LV5 (EF‐1a/GFP/Puro/Amp) lentiviral vector carrying human DJ‐1 (Sangon Biotech, Shanghai China). Cells transfected with the empty vector (Mock) were used as the negative control. To generate stable DJ‐1 knockdown cells, cells were transfected with the LV3 (pGLVH1/GFP/Puro) lentiviral vector (Sangon Biotech) carrying self‐complementary hairpin DNA fragments that could generate DJ‐1‐specific shRNA or control (scrambled) shRNA. The following sequences were used Sh*DJ‐1*, 5′‐GGAGGTCATTACACCTACTCT‐3′, and scrambled, 5′‐TTCTCCGAACGTGTCACGT‐3′. Fresh culture medium containing 4 *μ*g/mL of puromycin was added to select stable puromycin‐resistant cells.

### Reverse transcription and quantitative real‐time PCR

Total RNA was extracted from cells and 27 CRC samples using the RNAiso reagent (Takara, Kusatsu, Shiga Japan) according to the manufacturer's protocol, and cDNA was synthesized by reverse transcription quantitative real‐time PCR performed using the One Step SYBR kit (Takara) and the following primers: DJ‐1 forward, 5′‐TGGCTAAAGGAGCAGAGGAA‐3′, and reverse, 5‐ATGACCACATCACGGCTACA‐3′; GAPDH forward, 5′‐TGTTCGTCATGGGGTGAAC‐3′, and reverse, 5′‐ATGGCATGGACTGTGGTCAT‐3′; and PTEN forward: 5′‐TTT‐GAAGACCATAACCCACCAC‐3′, and reverse, 5′‐ATTACACCAGTTCGTCCCTTTC‐3′.

### Western blotting

Cells or tissues were washed twice with ice‐cold PBS and were lysed with RIPA buffer (Beyotime Biotech, Shanghai China). Aliquots of cell lysates containing 20 *μ*g of protein were separated on SDS‐polyacrylamide gels and were transferred to PVDF membranes. The membranes were blocked in PBST buffer containing 5% nonfat milk and were incubated at 4°C overnight with primary antibodies against DJ‐1 (1:100; Abcam, Cambridge UK), PTEN (1:1000; Abcam), AKT (1:1000; CST), P‐AKT^s473^ (1:1000; CST), and GAPDH (1:1000; CST) and then with secondary horseradish peroxidase (HRP)‐conjugated goat anti‐rabbit IgG (1:5,000; Sigma). The protein bands were visualized using the ECL kit (Beyotime Biotech Shanghai, China).

### Invasion and migration assays

For the migration assay, CRC cells were plated at the density of 2 × 10^4^ cells/well in serum‐free DMEM in the upper chamber of 24‐well Transwell plates containing 8.0‐*μ*m‐pore Millicell inserts, while the lower chambers were filled with DMEM supplemented with 10% FBS as a chemotaxis agent. For the invasion assay, Millicell inserts were coated with 1 mg/mL of Matrigel (BD Biosciences San Jose, CA). After 24 h of incubation, the inserts were removed from the plates, fixed in 4% paraformaldehyde, and stained with Crystal Violet Staining Solution (Beyotime). Cells invaded/migrated to the lower membrane surface were counted under a light microscope at ×200 magnification in at least four randomly selected fields, and the average number of cells per field was calculated. All the experiments were performed in triplicate.

### Cell proliferation assay

SW480 and HCT116 cells were seeded into 96‐well plates at the concentration of 1000 and were cultured for 24, 48, 72, and 96 h. At the indicated intervals, 20 mL of Cell Counting Kit‐8 (Beyotime) was added to each well for 1 h at 37°C, and the absorbance at 490 nm was measured using a Thermo Multiskan Spectrum Reader (Thermo Fisher Scientific Waltham, MA).

### Immunohistochemistry and scoring

Immunohistochemistry (IHC) detection of DJ‐1 was performed using the Dako Envision FLEX+ system. Tissue samples embedded in paraffin were sectioned, deparaffinized, and subjected to antigen retrieval performed in citrate buffer (pH = 6.0) for 15 min using a microwave oven. Slides were cooled to room temperature, washed in PBS, and incubated at 4°C overnight with the DJ‐1 antibody (1:25; Abcam) and then with a secondary HRP‐conjugated antibody (Dako Santa Clara, CA) at 37°C for 30 min. Sections were stained with 3‐diaminobenzidine (DAB) for 2 min and scored for the extent of DJ‐1 expression using the following system: 0, 0–5% DJ‐1‐positive cells; 1, <25% positive cells; 2, 25–50% positive cells; 3, 50–75% positive cells; and 4, 75–100% positive cells. The staining intensity was scored as follows: 0, no positive staining; 1, weak staining; 2, moderate staining; and 3, strong staining. The final scores were obtained by multiplying the extent scores by intensity scores (0, 1, 2, 3, 4, 6 8 9 or 12) and analysis using the statistical X‐tile software with score of 8 as the cut‐off value 12.

### Tumor implantation

All animal experiments were approved by the Institutional Animal Care and Use Committee of Xinqiao Hospital, TMMU. In total, 1 × 10^6^ DJ‐1 knockdown cells or control cells were suspended in 100 *μ*L of PBS and were injected into 5‐week‐old female nude mice. The size of the resultant tumors was measured every 7 days for a month using a Vernier caliper, and the calculated volume = shortest diameter^2^ × longest diameter/2 at seven‐day intervals posttransplantation. The animals were sacrificed one month after cell implantation, and subcutaneous xenografts were analyzed by Western blotting.

### Statistical analysis

Statistical analyses were conducted using SPSS software 19.0. The correlation between DJ‐1 expression and clinicopathological features was accessed by Pearson's chi‐squared test. Survival curves were obtained by the Kaplan–Meier method, and comparisons were made by the log‐rank test. Paired Student's *t*‐test for two groups and one‐way ANOVA for multiple groups were used. To investigate independent prognostic factors, variables that were significantly correlated with the follow‐up information from the univariate and multivariate analyses were used, and the Cox regression model was performed for the analysis. The difference was considered significant at a *P* value <0.05 (assigned as *), less than 0.01 (assigned as **), or less than 0.001 (assigned as ***). All experiments were carried out at least three times in triplicate.

## Results

### DJ‐1 is unregulated in human CRC tissues

**Figure 1:**
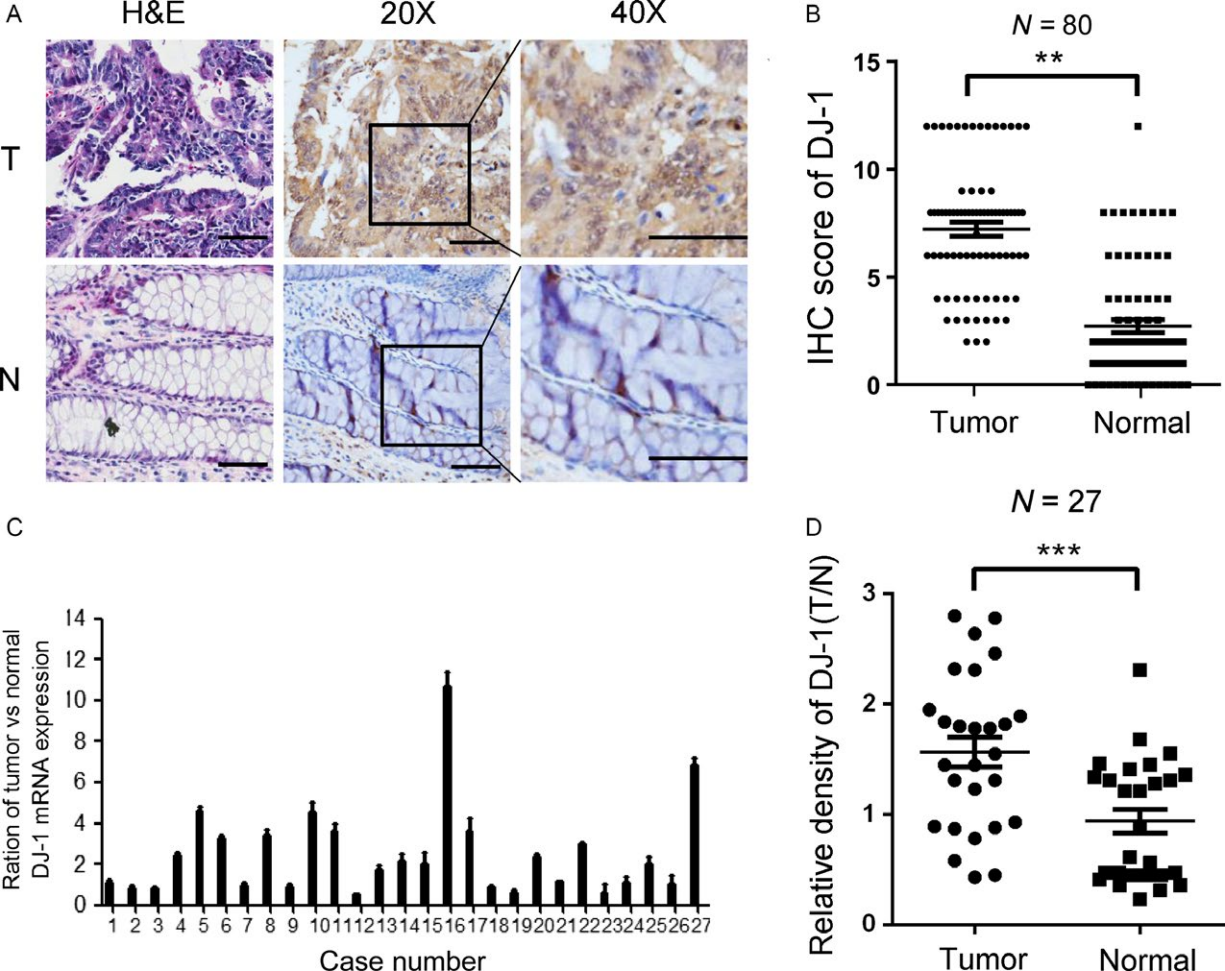
DJ‐1 is highly expressed in human CRC tissues. (A) Representative images of HE staining and DJ‐1 staining (brown color) in CRC samples (T) and normal colon tissue (N) (scale bar = 50 *μ*m). (B) IHC scores of tumors and adjacent normal tissues from 80 paired CRC specimens. (C) mRNA expression of DJ‐1 in adjacent normal tissues and matching cancerous tissues in 27 CRC specimens. (D) Expression ratios (DJ‐1/GAPDH) of each paired sample in 27 CRC specimens.

To explore the potential function of DJ‐1 in CRC, we first investigated its expression pattern in 80 pairs of CRC specimens and corresponding neighboring noncancerous tissues. In both tissue groups, DJ‐1 was mostly localized in the cell cytoplasm, and the proportion of cells with high DJ‐1 expression was significantly higher in CRC tissues (56%, 56 of 100) than in the adjacent normal tissues (11.25%, 9 of 80) (Fig. 1A and B and Table 1). This expression pattern was confirmed by qRT‐PCR analyses of 27 pairs of CRC tissues and matched normal colon tissues, which revealed that DJ‐1 mRNA (Fig. 1C) was upregulated in tumors. Additionally, DJ‐1 protein was expressed significantly higher in 27 CRC tissues than in corresponding normal tissues (Figs 1D and S1). Taken together, these results indicated that DJ‐1 expression was significantly higher in CRC specimens than in the adjacent noncancerous colon tissues.

**Table 1:** DJ‐1 expression in CRC and adjacent normal mucosa

	DJ‐1(Low)	DJ‐1(high)	*P* value
Carcinoma	44	56	<0.001
Adjacent normal mucosa	71	9	

### High DJ‐1 expression in CRC is associated with the TNM stage and poor prognosis

**Figure 2:**
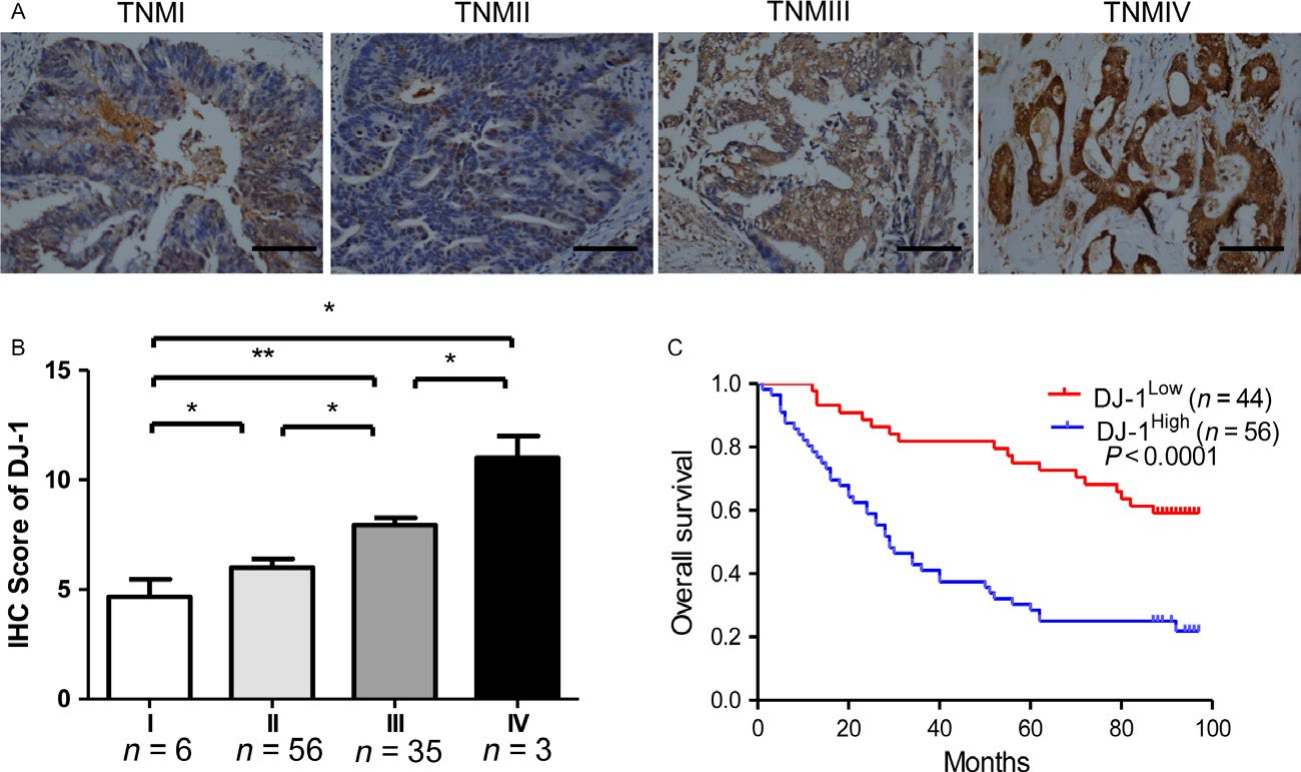
DJ‐1 protein expression and its prognostic value in CRC. (A) Representative images of DJ‐1 staining in tumors and normal colorectal mucosa of paired CRC specimens from patients with different TNM stages (scale bar = 200 *μ*m). (B) IHC scores of DJ‐1 expression in CRC specimens of all cancer stages. DJ‐1 levels were significantly higher in tumors with a high TNM stage than in those with a low TNM stage. (C) Kaplan–Meier survival analysis of 100 CRC patients showing that patients with high DJ‐1 expression (*n *= 56) had a shorter overall survival than those with low DJ‐1 expression (*n *= 44) (*P *< 0.0001).

We next analyzed the association of DJ‐1 protein expression with the clinical pathological characteristics of patients with CRC. The clinicopathological characteristics are shown in Table 2. The results indicated that DJ‐1 expression was higher in patients with advanced disease stage than in those with early‐stage cancer (Figs 2A and 1B) and was correlated with tumor depth (*P *= 0.026) and lymphatic metastasis (*P *= 0.021). However, there was no significant difference in DJ‐1 expression between patients with well‐differentiated and moderately differentiated tumors (Table 3). Kaplan–Meier survival curves showed that the OS for patients with high DJ‐1 levels was significantly shorter than that for those with low DJ‐1 levels in tumors (Fig. 2C, *P *< 0.001). Univariate and multivariate analyses showed that advanced TNM stage and high DJ‐1 expression were significantly related to an unfavorable OS, indicating that DJ‐1 is an independent predictive factor of poor prognosis (HR = 3.265; 95% CI: 1.779–5.992; *P *= 0.000; Table 4). Thus, DJ‐1 could serve as a prognostic biomarker and a risk factor in patients with CRC.

**Table 2:** The clinical features of the CRC specimens used in this study

Feature	WHO grade		
	I (*n *=* *5)	II (*n *=* *45)	III (*n *=* *50)
Gender			
Male	4	23	28
Female	1	22	22
Age at diagnosis (year, mean ± SD)	74.80 ± 14.02	69.98 ± 10.10	65.78 ± 13.38
<50	0	1	5
≥50	5	44	45
Invasive depth			
Submucosa	1	1	0
Muscular layer	1	2	20
Serous layer	0	33	30
Whole layer	3	9	10
Location			
Sigmoid colon	2	14	9
Ascending colon	0	9	16
Transverse colon	1	11	13
Descending colon	2	11	10

**Table 3:** The relationship between DJ‐1 expression and clinicopathological features of patients with CRC

Feature	DJ‐1		
	High (*n *= 56)	Low (*n *= 44)	*P* value
Gender			
Male	27 (48.2%)	28 (63.6%)	0.124
Female	29 (51.8%)	16 (36.4%)	
Age at diagnosis (Year, mean ± SD)	68.44 ± 14.20	67.51 ± 11.54	0.394
<50	5 (8.93%)	2 (4.56%)	
≥50	51 (91.07%)	42 (95.44%)	
Location			
Transverse colon	17 (30.36%)	8 (18.18%)	0.025*
Ascending colon	12 (21.43%)	13 (29.56%)	
Descending colon	13 (23.21%)	10 (22.73%)	
Sigmoid colon	14 (25.00%)	13 (29.54%)	
T stage			
T_1‐3_	43 (76.79%)	41 (93.18%)	0.026*
T_4_	13 (23.21%)	3 (6.82%)	
N stage			
N0	28 (50.00%)	32 (72.73%)	0.021*
N1‐2	28 (50.00%)	12 (17.17%)	
TNM			
*Ι*+Π	30 (53.57%)	32 (72.73%)	0.048*
*ΙΙΙ*+IV	26 (46.43%)	12 (17.17%)	
Histological grade			
Well	3 (5.36%)	2 (4.56%)	0.789
Moderate	27 (48.21%)	17 (38.64%)	
Poor	26 (46.42%)	25 (56.80%)	

*P* value <0.05

**Table 4:** Univariate and Multivariate analysis for overall survival in CRC

Factors	Univariate		Multivariate	
	HR (95%CI)	*P* value	HR (95%CI)	*P* value
Gender	1.088 (0.657–1.801)	0.743	0.696 (0.403–1.200)	0.192
Age	1.019 (0.996–1.042)	0.839	1.017 (0.995–1.039)	0.134
Location	0.898 (0.725–1.114)	0.328	0.918 (0.735–1.145)	0.447
DJ‐1 expression	3.124 (1.791–5.449)	0.000^a^	3.265 (1.779–5.992)	0.000^a^
Grade	1.471 (0.938–2.308)	0.093	1.784 (1.066–2.986)	0.028^a^
TNM Stage	2.993 (1.916–4.676)	0.000^a^	2.574 (1.599–4.144)	0.000^a^

a *P* value <0.05

### DJ‐1 promotes the migration, invasion, and proliferation of CRC cells *in vitro*

**Figure 3:**
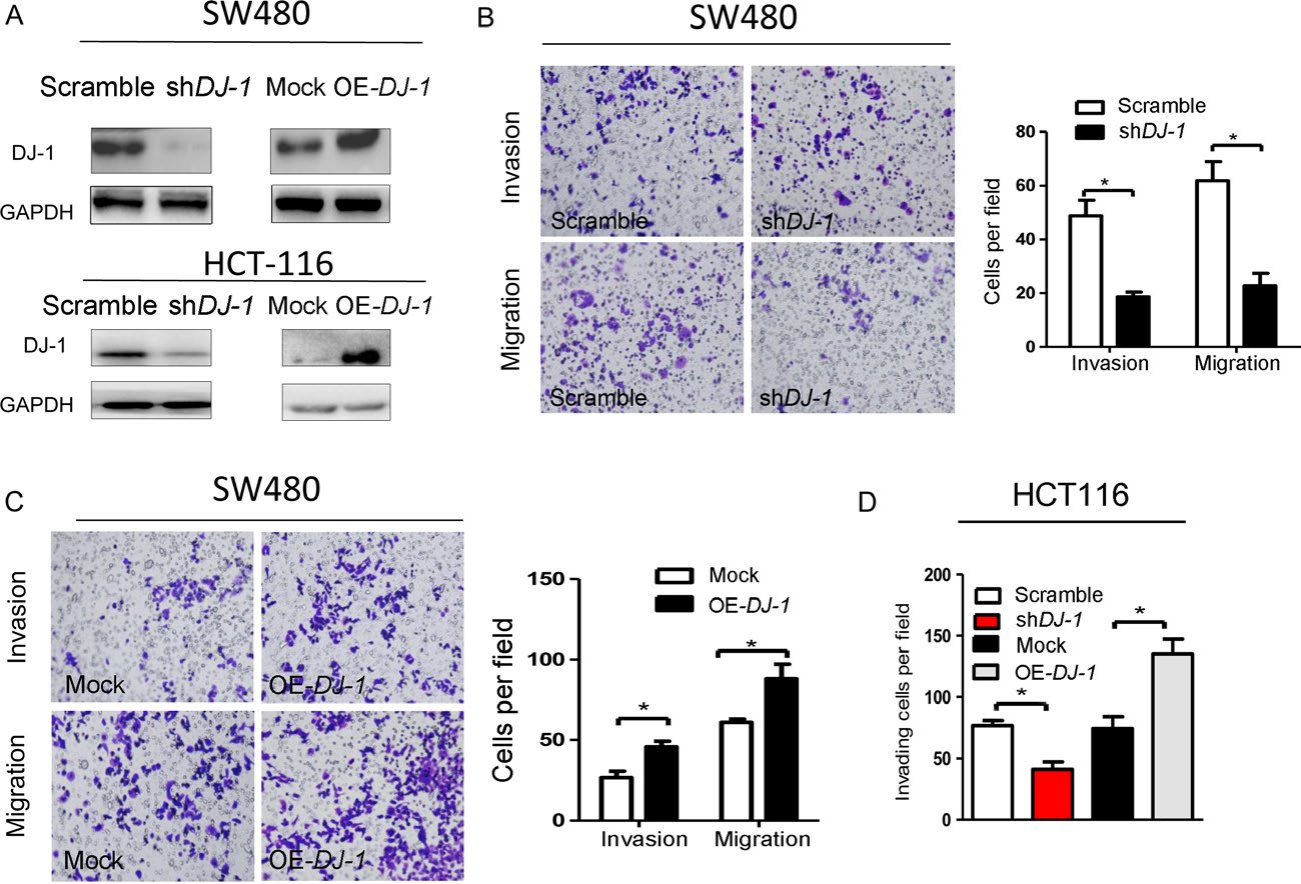
DJ‐1 promotes the invasion of colorectal cancer cells in vitro. SW480 and HCT‐116 cells were transduced with lentiviral vectors to express *DJ‐1*‐specific shRNA or overexpress *DJ‐1*; scrambled shRNA and empty vector were used as negative controls. (A) Western blotting analysis of DJ‐1 in scrambled, sh‐*DJ*‐1, mock and OE‐*DJ*‐1 in SW480 and HCT‐116 cells. (B–C) Cell migration and invasion as analyzed by the Transwell assay in DJ‐1‐knockdown cells (B) and DJ‐1‐overexpressing (C) SW480 cells. Scale bar = 50 *μ*m. The values represent the mobility of shCtrl‐, sh*DJ‐1‐,* and mock‐OE‐*DJ‐1*‐SW480 cells as detected by migration and invasion assays. *P <* *0.05; **P <* *0.01. (D) Cell migration and invasion analyzed by the Transwell assay in DJ‐1‐knockdown and DJ‐1‐overexpressing HCT‐116 cells. *P <* *0.05; **P <* *0.01. Experiments in B–D were performed at least in triplicate, and the data are presented as the means ± SD.

To examine the underlying role of DJ‐1 in CRC malignancy, we used colon cancer cell lines SW480 and HCT‐116 with different levels of DJ‐1 expression to generate DJ‐1‐knockdown and DJ‐1‐overexpressing cells (Fig. 3A). The results revealed that DJ‐1‐knockdown SW480 cells invaded and migrated into the lower chamber slower than control cells (Fig. 3B, *P *< 0.05), Furthermore, the number of invading and migrating CRC cells transfected with OE‐*DJ‐1* was increased compared with that of mock cells (Fig. 3C, *P *< 0.05). These observations were confirmed in HCT‐116 cells (Fig. 3D).

**Figure 4:**
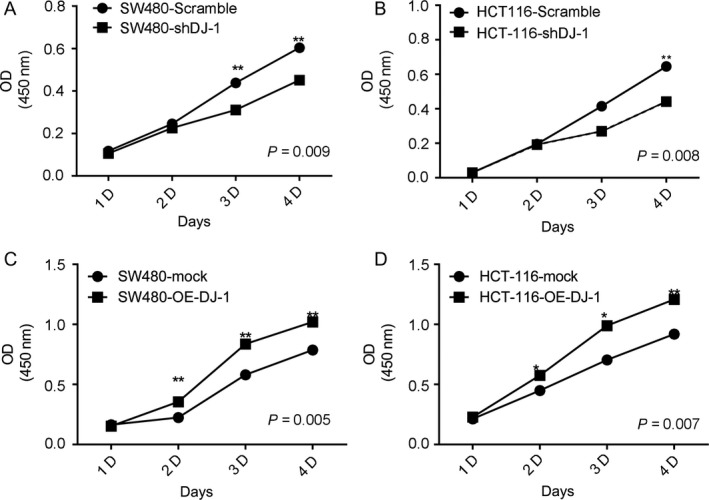
DJ‐1 promotes the proliferation of colorectal cancer cells in vitro. (A–B) Growth curves of DJ‐1‐knockdown and control SW480 cells (A) and HCT116 cells (B). (C–D) Growth curves of DJ‐1‐overexpressing and control SW480 cells (C) and HCT‐116 cells (D).

We next evaluated the effect of DJ‐1 on the growth of colon cancer cells and found that DJ‐1 knockdown reduced, whereas DJ‐1 overexpression induced, cell proliferation (Fig. 4A–D).

### DJ‐1 promotes CRC progression by inhibiting PTEN expression and activating AKT

**Figure 5:**
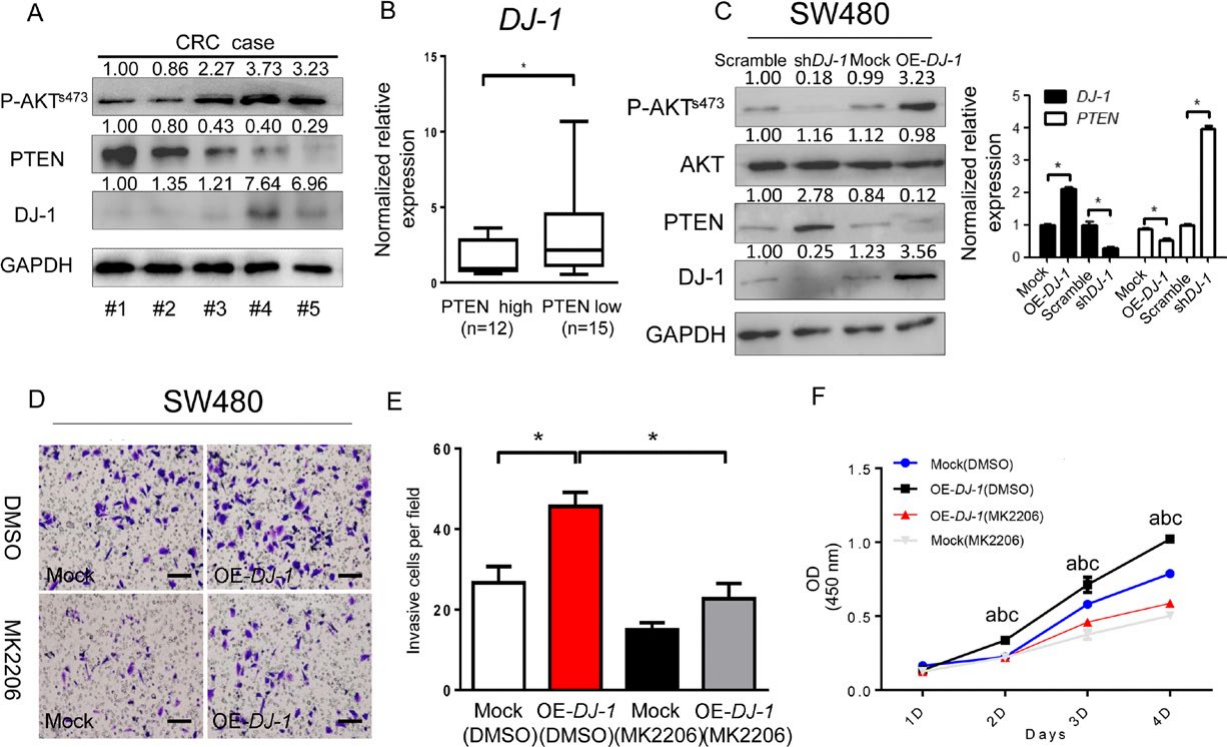
DJ‐1 promotes the invasion and proliferation of colorectal cancer cells by inhibiting PTEN expression and activating the AKT pathway. (A) DJ‐1, PTEN, and P‐AKT ^s473^ were detected by WB in five fresh human CRC samples. (B) DJ‐1 mRNA expression levels in the PTEN‐high group and PTEN‐low group. PTEN‐high group: PTEN mRNA fold change (tumor/normal) >1.5; PTEN‐low group: PTEN mRNA fold change (tumor/normal) ≤1.5; *P <* *0.05; (C) Analyses of the levels of PTEN and P‐AKT ^s473^ protein (*left panel*) and mRNA (*right panel*) in scrambled control cells, sh‐*DJ‐1‐* SW480 cells, mock cells, and OE‐*DJ‐1*‐SW480 cells. *P <* *0.05; (D–E) Invasion of SW480 cells treated with 1 *μ*mol/L of MK2206. Representative images (D) and numbers (E) of invaded cells (scale bar = 50 *μ*m). (F) Cell growth as assessed by the cell proliferation assay. Growth curves from the indicated cells as assessed by the cell proliferation assay. a, b, and c denote significance among different groups. a: (mock vs. OE‐DJ‐1), *P *< 0.05; b: (DMSO þ mock vs. MK2206 þ mock), *P *< 0.01; c: (OE‐DJ‐1þ MK2206 vs. OE‐DJ‐1 þ MK2206), *P *< 0.01; *P <* *0.05; **P <* *0.01. Experiments in B‐E were performed at least in triplicate, and the data are presented as the means ± SD.

A previous study has demonstrated that DJ‐1 could antagonize the tumor suppressor PTEN to inhibit the activity of the PTEN gene and finally promote the proliferation of tumor cells 13. Therefore, we next examined whether activation of the PTEN/AKT pathway was the mechanism underlying the oncogenic effects of DJ‐1 in CRC. Western blotting analysis of protein extracts from five fresh CRC samples revealed an inverse correlation of PTEN expression with the protein levels of DJ‐1 and P‐AKT^s473^ (Fig. 5A). Furthermore, DJ‐1 mRNA was expressed higher in the PTEN‐low group than in the PTEN‐high group (Fig. 5B). Consistent with this observation, PTEN expression was significantly increased in DJ‐1‐knockdown cells compared with that in scrambled cells, whereas DJ‐1‐overexpression resulted in the reduction of PTEN protein levels (*Left panel*) and mRNA (*Right panel*), as well as in the upregulation of AKT phosphorylation (L*eft panel*) (Figs 5C and S2). However, the inhibition of activity by MK2206 (Akt phosphorylation inhibitor) partially decreased the positive effects of DJ‐1 on CRC cell invasion (Fig. 5D–E) and proliferation (Fig. 5F). These results suggest that DJ‐1 promoted CRC progression through the downregulation of PTEN expression and activation of the AKT signaling pathway.

### The DJ‐1/PTEN/AKT signaling pathway accelerates CRC progression *in vivo*

**Figure 6:**
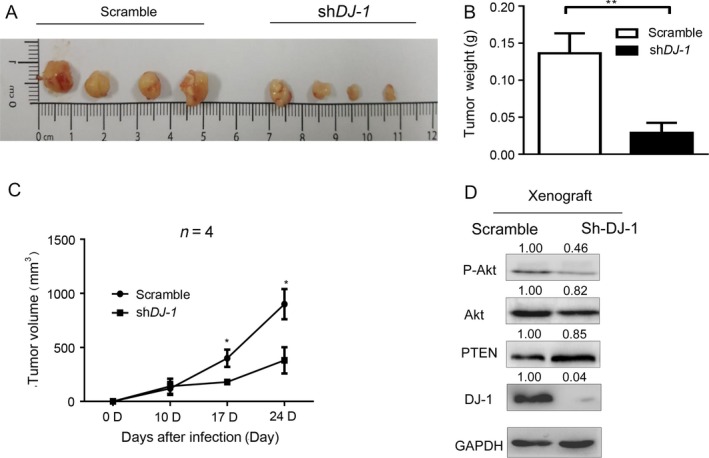
DJ‐1 accelerates CRC progression in vivo. (A) Subcutaneous implantation of SW480 cells in nude mice (1 × 10^6^ cells/mice). (B–C) Quantitative analysis of the weight (B) and volume (C) of xenograft tumors *P *< 0.01; *P < 0.05. (D) Expression of DJ‐1, PTEN, and P‐AKT ^s473^ in xenograft tumors derived from DJ‐1‐knockdown and control cells.

We then assessed the effect of DJ‐1 knockdown on the oncogenic potential of SW480 cells using a subcutaneous xenograft model in nude mice. The xenograft tumors formed by implanting DJ‐1‐knockdown cells had an average smaller weight than those formed by control cells (Fig. 6A, B) and demonstrated slower growth (Fig. 6C). Furthermore, the expression of PTEN was increased, whereas that of DJ‐1 and P‐AKT^s473^ decreased in tumors derived from DJ‐1‐knockdown cells compared to those derived from control cells (Fig. 6D), suggesting that the DJ‐1/PTEN/AKT pathway is involved in the progression of colon cancer cells in vivo.

## Discussion

It is established that unlimited proliferation and migration of tumor cells are responsible for the initiation, invasion, and metastasis of cancer, including CRC, defining its progression and recurrence 14. Herein, we revealed, for the first time, a role of DJ‐1 in the regulation of invasion and proliferation of CRC cells. DJ‐1 expression was correlated with the migration, invasion, and growth of colon cancer cells, suggesting that DJ‐1 is a critical mediator of CRC progression. Furthermore, we analyzed 100 CRC patients with follow‐up information and found that DJ‐1‐high patients with CRC showed a shorter overall survival (OS) than DJ‐1‐low patients. Univariate and multivariate analyses showed that DJ‐1 could be an independent prognostic factor for the OS of patients with CRC. To the best of our knowledge, this study was the first to investigate the relationship between DJ‐1 expression and CRC development in a cohort of patients followed up for a long time.

DJ‐1, a 20‐kDa protein conserved among prokaryotic and eukaryotic cells, has multiple biological functions 15. *DJ‐1* mutation can suppress the neuronal loss related to Parkinson's disease 16 and in the progression of sepsis and inflammation 17. Furthermore, DJ‐1 can act as an antioxidant by attenuating oxidative stress, supporting the function of mitochondria during ischemia/reperfusion injury 18. It seems that DJ‐1 protein is closely related to chronic inflammation. In fact, the prognostic significance of DJ‐1 expression has been suggested in breast cancer 19, and DJ‐I deficiency inhibits the activation of IL‐6/STAT3 signaling in mice with hepatocellular carcinoma 20. These results indicate that DJ‐1 plays a role in regulating the inflammation status, including that in cancer. Furthermore, DJ‐1 deficiency decreased the CD4+:CD8+ T‐cell ratio and promoted the development of regulatory T cells 21 that is associated with a positive outcome in CRC 22, suggesting that DJ‐1 promotes tumor progression by inhibiting tumor immunology in CRC.

Given that the secretion profile of proinflammatory cytokines and activity of immune cells are closely correlated with CRC progression, we hypothesized that DJ‐1 should play a role in CRC. Indeed, in our study, we found that DJ‐1 expression was upregulated in colorectal tumors compared with that in noncancerous colon tissues. Higher expression was directly correlated with advanced CRC stage and a poor prognosis. Thus, DJ‐1 may act as a proinflammatory factor in CRC initiation and progression.

Furthermore, DJ‐1 was shown to perform transcriptional regulation in neuronal cells 23 and nonsmall cell lung carcinoma cells where it activated the PI3K/AKT pathway 13. The tumor suppressor gene phosphatase and tensin homolog deleted from chromosome 10 (PTEN) is the key negative regulator of PI3K signaling 24, and its mutation is closely linked to the development, progression, and prognosis of CRC 25. Epigenetic inactivation of PTEN has recently been reported in various human tumors, but the transcriptional regulation of PTEN has been rarely reported 26. In our study, we found that PTEN mRNA is significantly increased in sh*DJ‐1* colon cells compared with scrambled control cells, whereas PTEN mRNA was decreased in OE‐*DJ‐1* colon cells more than in mock cells, indicating that DJ‐1 may participate in regulating PTEN transcriptionally. Furthermore, DJ‐1 expression was negatively correlated with PTEN expression in tumor specimens in a previous study 5, but the underlying mechanisms of PTEN/AKT and DJ‐1 have been rarely reported in cancers, especially in CRC. In the current study, we found that CRC tissue samples with high levels of DJ‐1 or P‐AKT^s473^ lacked PTEN protein, whereas DJ‐1‐knockdown colon cancer cells demonstrated an increase in PTEN expression that, in contrast, was decreased in DJ‐1‐overexpressing cells compared with that in the control. Given that DJ‐1 promoted CRC cell invasion and proliferation and correlated with advanced disease stage and a poor prognosis, these findings suggest that DJ‐1 exerted its oncogenic effects by regulating the PTEN/AKT signaling pathway. It is known that the PTEN/AKT pathway plays a crucial role in the formation of CRC, but targeting the PTEN/AKT signaling pathway drug rarely improves the 5‐year survival rate 27. Our research provides a new strategy in inhibiting the PTEN/AKT pathway. Furthermore, DJ‐1 interacts with the cytoplasmic C‐terminal of HER3 to accelerate tumor progression 28. Thus, the mechanisms underlying DJ‐1‐mediated CRC progression are complex and may explain why using the Akt inhibitor does not completely prevent cell growth in vitro.

There are still some limitations in this study. Firstly, the sample size to detect DJ‐1 protein expression in the tumors of patients with CRC is small (*n *= 100), and the multivariate analysis results for overall survival in CRC (Table 4) showed that the value of HR for DJ‐1 is higher than that of TNM, indicating that DJ‐1 has a controversial high sensitivity; therefore, more samples are needed to support our results that the high expression of DJ‐1 could predict the poor prognosis. Secondly, because the CRC sample size with Stage IV is too small (*n *= 3), it is necessary to further explore the relationship between TNM stage and DJ‐1 expression. Thirdly, although we have presented a mixture of preclinical and clinical data, the evidence that DJ‐1 acts as a potential prognosis biomarker and therapeutic target is valuable. Prospective clinical investigations are needed to generate data to confirm this clinical application.

In summary, our study revealed a novel role of DJ‐1 as an oncogene in CRC that stimulates the migration, invasion, and proliferation of CRC cells through the downregulation of PTEN. Therefore, our research provides new insights into the mechanism responsible for CRC development and suggests DJ‐1 as a promising therapeutic target in CRC.

## Conflict of Interest

None.

## Supporting information


**Figure S1.** All images of 27 paired CRC tissues by Western blotting.

**Figure S2.** Relative density of p‐AKTs473, AKT, PTEN, and DJ‐1 in scrambled, sh*DJ‐1*, mock and OE*DJ‐1* SW480 cells.
