# Supplementary figures and images for: High expression of DJ‐1 promotes growth and invasion via the PTEN‐AKT pathway and predicts a poor prognosis in colorectal cancer
Source: Cancer Med. 2018 Feb 14;7(3):809–19. doi: 10.1002/cam4.1325 (PMC5852339; doi:10.1002/cam4.1325)

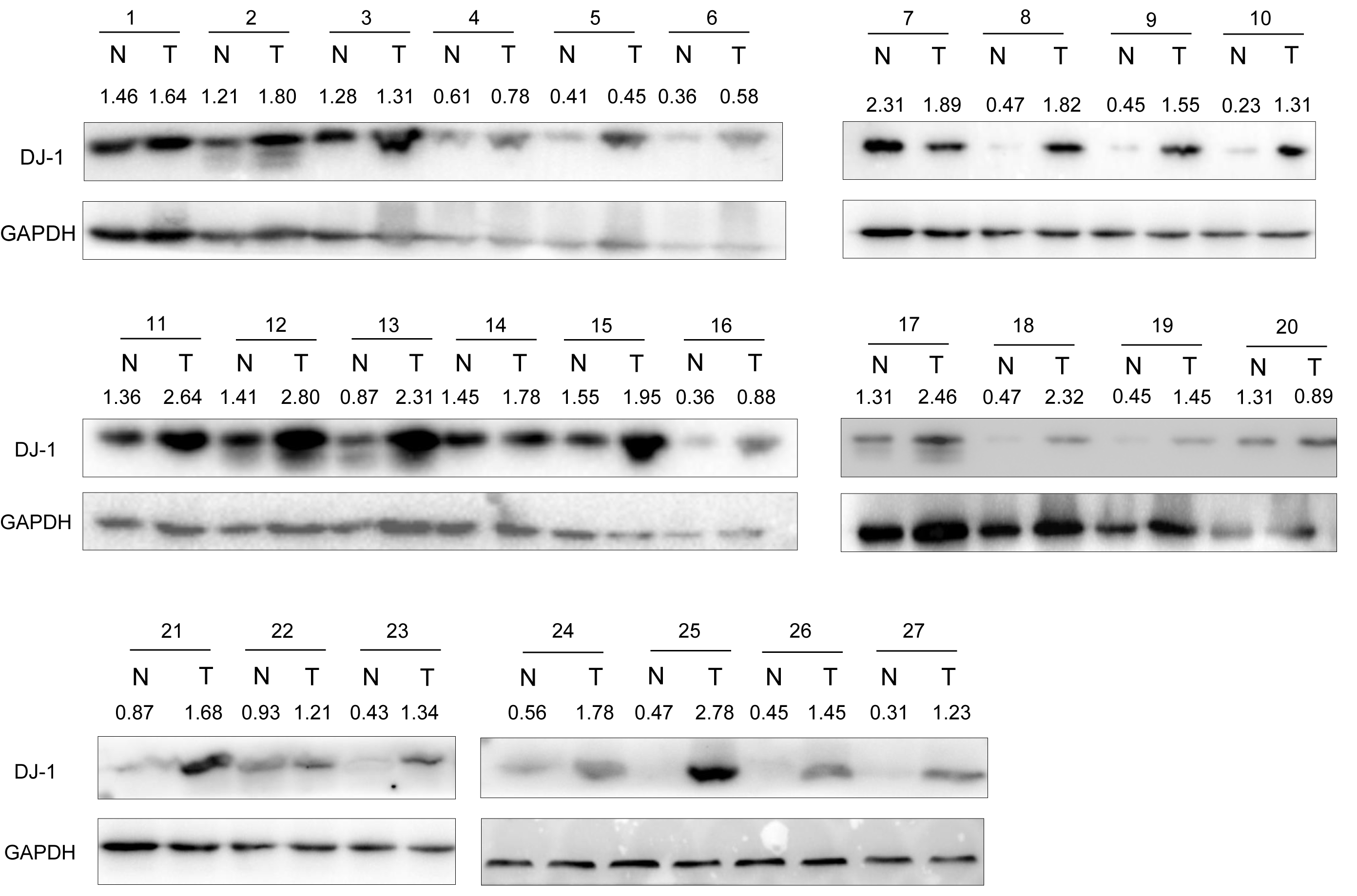

Supplement: Supplementary file 1 — Figure S1. All images of 27 paired CRC tissues by Western blotting. [file CAM4-7-809-s001.tif]

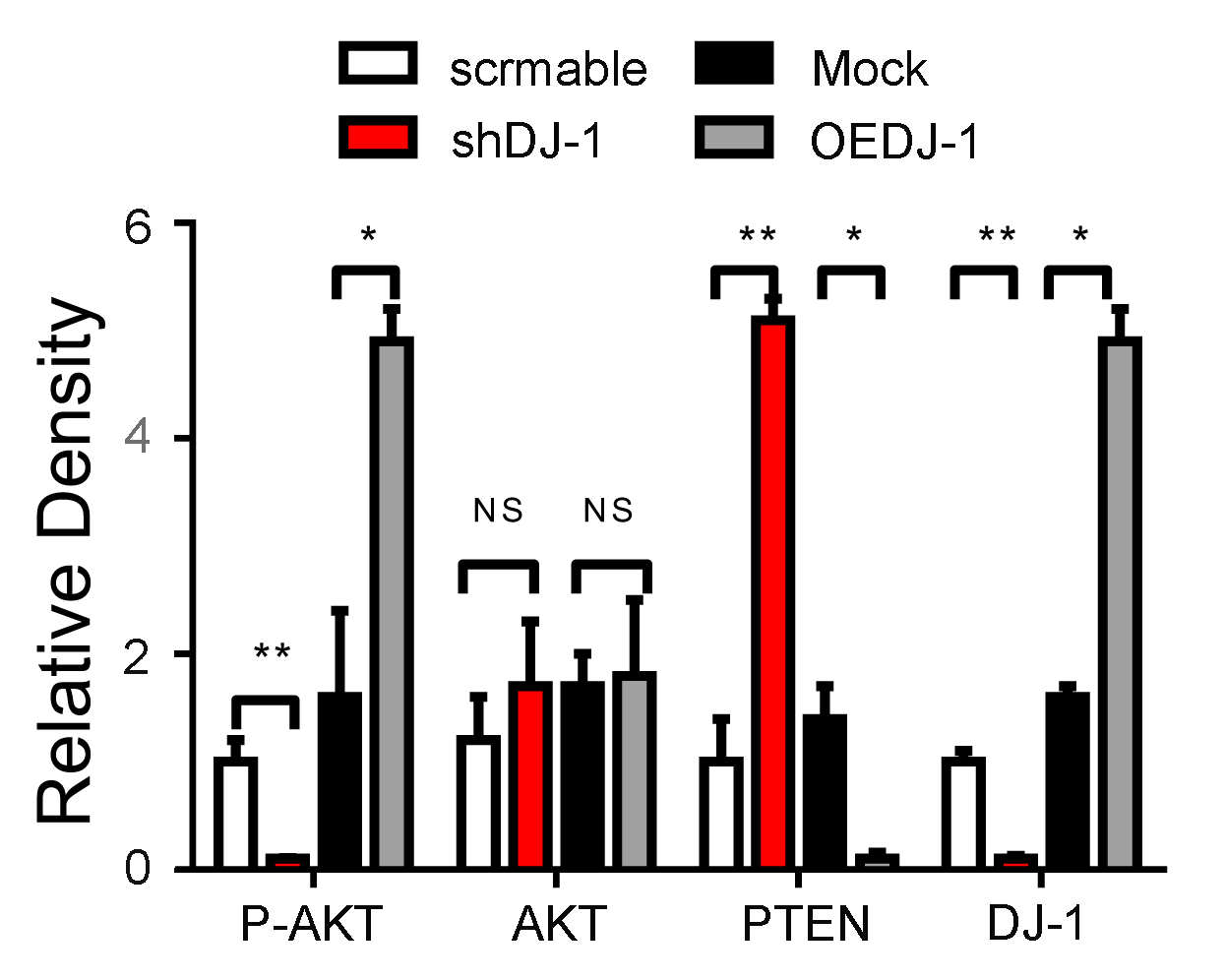

Supplement: Supplementary file 2 — Figure S2. Relative density of p‐AKTs473, AKT, PTEN, and DJ‐1 in scrambled, shDJ‐1, mock and OEDJ‐1 SW480 cells. [file CAM4-7-809-s002.tif]
